# Neuropsychological and Emotional Functioning in Patients with Cushing's Syndrome

**DOI:** 10.1155/2020/4064370

**Published:** 2020-08-07

**Authors:** Sabrina Na, Mary A. Fernandes, Adriana G. Ioachimescu, Suzanne Penna

**Affiliations:** ^1^Division of Rehabilitation Neuropsychology, Department of Rehabilitation Medicine, Emory University School of Medicine, USA; ^2^Department of Psychology, Georgia State University, USA; ^3^Division of Endocrinology Metabolism and Lipids, Department of Medicine, Emory University School of Medicine, USA; ^4^Department of Neurosurgery, Emory University School of Medicine, USA

## Abstract

Patients with Cushing's syndrome (CS) frequently report impairments in cognitive and emotional functioning. Given neuroimaging research that implicates alterations in structure and function in the brain in this population, goals of this study were to investigate neuropsychological and emotional functioning, with particular emphasis on complex attention and memory. In a clinical sample of 18 adults with CS referred for neuropsychological evaluation (age 41.6 ± 10.6, 72% Caucasian), patients' most common subjective complaints were in attention and increased irritability. On objective testing, patients exhibited significant declines in the consistency of their sustained attention and visual-spatial functioning compared to normative peers. Patients exhibited on average significantly reduced initial learning following first exposure to visual and verbal stimuli but intact retention of information learned. Patients with CS endorsed highly elevated levels of somatization, depression, and anxiety, and 59% of them scored in the clinically elevated range for somatization and depressive symptomatology. Exploratory analyses suggested that the 11 patients with active Cushing's exhibited lower processing speed, poorer sustained attention, naming, and cognitive flexibility compared to the 7 patients who achieved biochemical remission. Patients with active Cushing's also reported higher levels of somatization and anxiety compared to patients in biochemical remission. Overall, this study provides new insights into complex attention and memory deficits in patients with CS and concern regarding cognitive and emotional issues despite resolution of hypercortisolism. Our study opens several avenues for further exploration.

## 1. Introduction

Cushing's syndrome (CS) is characterized by endogenous hypercortisolism as a result of a pituitary, adrenal, or neuroendocrine tumor. Cushing's disease (CD) is the most common form of CS which occurs in context of hypersecretion of the adrenocorticotropic hormone (ACTH) by a pituitary tumor, with incidence of 1.2 to 2.4 per million per year [1]. Elevated levels of cortisol exert system-wide effects that result in various clinical features (e.g., rounded face, centripetal fat accumulation, high blood pressure, and skin changes). The condition is associated with significant medical comorbidities, including hypertension, hyperglycemia, cardiovascular disease, infections, and fractures [2]. Primary treatment of Cushing's disease is pituitary surgery by an expert neurosurgeon which results in most patients going into biochemical remission [3]. For patients who experience persistent or recurrent hypercortisolism postoperatively, pituitary radiation, bilateral adrenalectomy, and medications can be used. Primary treatment of adrenal Cushing's syndrome is adrenalectomy. Both pituitary and adrenal procedures are followed by a phase of corticoadrenal insufficiency (i.e., low cortisol) which is reversible after a few months or years (unless the adrenal procedure is bilateral adrenalectomy).

In addition to medical comorbidities, CS patients often experience declines in quality of life, cognitive dysfunction, and disruptions in psychological functioning. Patients with active Cushing's syndrome report diminished school and work performance, sleep difficulties, and difficulties with interpersonal relationships [4]. Neurocognitive impairments have been documented in the domains of memory, concentration, visuospatial functioning, and language functioning [4–7]. Patients with active CS also demonstrate significant emotional changes due to the hypersecretion of cortisol, notably symptoms of depression, anxiety, and mania [8–12].

A small but burgeoning area of research has also found that these difficulties may persist for patients who have achieved biochemical remission for several years [13]. In one study comparing patients with CS to patients with treated nonfunctioning adenomas, CS patients in remission reported increased irritability, negative affect, somatic arousal, maladaptive personality traits, and lower positive affect [14]. Deficits in cognitive domains appear to similarly linger following remission. In one study, among CS patients who were in remission for an average of three years, deficits were observed in domains of attention, visuospatial processing, reasoning, fluency, and memory [6].

Prior research studies have emphasized the study of memory, both due to patient complaints of memory loss and given that the hippocampus is particularly sensitive to cortisol [15]. Memory is consistently shown to be affected in patients with CS in neuropsychological studies [4, 16], and reductions in hippocampal volumes have been documented in patients with active CS [4]. However, the pattern of memory deficits is somewhat unclear; studies typically provide a general domain or use summative scores to represent memory functioning which can obscure the types of memory deficits that patients are experiencing. As impaired performance on a memory measure may reflect problems at the level of encoding information, rapid forgetting, or problems with accurately retrieving information that they learned, it is important to determine the specific nature of memory deficits by evaluating each metric individually.

Furthermore, glucocorticoid receptors are diffusely present throughout the cortex [17], and targets of cortisol are widespread throughout the brain, suggesting that hypercortisolemia has more diffuse effects on cognition [18]. Indeed, neuroimaging studies investigating brain structure in patients in long-term remission have found persistent reductions in gray matter volume and decreased cortical thickness in prefrontal regions of the brain [19] even when hippocampal volumes have recovered [20]. Widespread reductions have been established in white matter volume and integrity [18, 21, 22]. Functional neuroimaging studies also have found alterations in the activity in the prefrontal cortex at rest and during tasks [23–25], as well as changes in functional connectivity within networks connecting the prefrontal cortex to the medial temporal lobe in patients with remitted CS [26]. These studies indicate a neurobiological basis for persistent cognitive deficits and suggest that emphasis should be placed on investigating cognitive functions that are supported by the prefrontal cortex, such as complex attention, learning/encoding of information, and executive functioning.

Notably, attention (particularly complex attention) is understudied in this literature. Prior research studies have been somewhat mixed with respect to whether attention deficits are present in patients with CS. This may be due to the use of simple digit span tasks as the primary measure of attention rather than more comprehensive measures. As disrupted attention can have downstream effects on the performance of many other neuropsychological measures, such as memory, it is essential to examine this domain with more sensitive measures that more accurately capture attention demands that patients face in everyday life. Complex attention processes involve an integrated activity of the network of regions in the brain, including prefrontal regions such as the anterior cingulate cortex and the dorsolateral prefrontal cortex [27]. These are precisely the regions implicated in structural and functional neuroimaging studies of CS [19, 25]. Therefore, we hypothesized that complex attention would be disrupted in patients with CS.

The purpose of this study was to examine neuropsychological and psychological functioning in patients with CS with a comprehensive battery of measures. Given the paucity of literature in this rare clinical group, our goals were to replicate prior findings and identify domains of cognitive deficits and psychological dysfunction in a sample of patients with CS. In particular, we were interested in exploring levels of somatization in patients, given clinical observations of somatic overconcern with cognitive functioning in patients with CS [28]. Another goal was to supplement current literature by investigating one highly understudied facet of complex attention: sustained attention. A final goal of this study was to characterize the specific nature of memory deficits through an in-depth examination of this domain.

## 2. Methods

### 2.1. Participants and Procedures

The participants of this retrospective study consisted of patients diagnosed with CS who were assessed at the outpatient neuropsychology clinic through the Division of Rehabilitation Neuropsychology at the Emory School of Medicine from 2015 to 2019. Diagnosis of Cushing's disease was based on abnormal screening tests (24-hour urine free cortisol, dexamethasone suppression and bedtime salivary cortisol) and localization tests confirming pituitary location (e.g., MRI of the pituitary gland and inferior petrosal sinus sampling). Patients were referred for a neuropsychological assessment as part of their standard medical care from the pituitary clinic due to patients' subjective complaints of cognitive and emotional changes. The evaluation began with an interview obtaining information regarding the patient's current cognitive and emotional complaints as well as relevant background information. Patients then underwent a comprehensive assessment which involved administration of a battery of measures designed to evaluate various domains of cognitive and emotional functioning. This information was summarized into a report and placed into the patient's medical record. After obtaining approval from the Institutional Review Board from the Emory University School of Medicine, a clinical database was created which included relevant demographic information regarding the patient, their current cognitive and psychological concerns, and objective performance on all measures administered during the evaluation. A dedicated pituitary endocrinologist (A.G.I.) reviewed the patient's medical records for information related to diagnosis, treatment(s) completed by the time of the assessment (e.g., dates/type of surgery, radiation treatment), and whether patients were in biochemical remission or active Cushing's syndrome at the time of their evaluation. Biochemical remission was defined as low or normal serum cortisol achieved as a result of surgery or radiation. Patients with normal serum cortisol levels had normal screening tests to exclude ongoing hypercortisolism.

### 2.2. Participant Characteristics

The ensuing sample consisted of 18 adults, 83% female, 72% Caucasian, mean age at evaluation 41.6 years ± 10.6. One patient had ACTH-independent adrenal Cushing's from bilateral macronodular hyperplasia, while the other 17 had an ACTH-secreting pituitary tumor (Cushing's disease).  Table 1 presents details regarding patients' characteristics and treatments at the time of testing.  Table 2 presents details regarding the patient's endocrinological situation at pretreatment and at the time of testing. As Cushing's syndrome can often be complicated by electrolyte disorders that can affect cognitive and psychological functioning, information regarding levels of potassium and glucose levels is also provided in Table 2. All patients with hypokalemia had normal potassium level at the time of testing. Among patients with diabetes, three had uncontrolled diabetes with HbA1c over 7%.

## 3. Measures

### 3.1. Neuropsychological Assessment

The neuropsychological battery consisted of the following measures (in parentheses) to assess these domains: simple auditory attention and working memory (digit Span: WAIS IV [30]), sustained attention (CPT-2 [31]), processing speed (Trails A and Coding subtest from WAIS-IV; [30]), visuospatial skills (judgment of line orientation and Benton facial recognition task; [32]), language (Boston Naming Test-II [33] and verbal fluency subtest from the Delis-Kaplan Executive Function System [34]), visuospatial learning and memory (BVMT-R), verbal learning and memory (CVLT-2^nd^ edition; [35]), and executive functioning (Trails B and verbal fluency category switching from the D-KEFS [34]). For descriptions of each measure and the metrics utilized, please refer to the Supplemental materials (S1).

All participants passed stand alone and/or embedded measures of performance validity; these measures were included to ensure adequate engagement on testing and that performances were valid reflections of current cognitive functioning. Performance on all measures was age-normed based on published normative data and converted to z-scores. For ease of interpretation, relevant performances were reverse scored such that on all neuropsychological measures, a more positive z-score indicated better performance.

### 3.2. Psychological Functioning

Patients were administered either the Minnesota Multiphasic Personality Inventory-2-Restructured Form (MMPI-2-rf; [36]) or the Personality Assessment Inventory (PAI; [37]), which are both validated measures of emotional functioning. Both measures include subscales that measure somatization (Scale 1: hypochondriasis scale on the MMPI-2-rf, somatic concerns scale on the PAI), depression (Scale 2: depression scale on the MMPI-2-rf, depression scale on the PAI), and anxiety (Scale 7: psychasthenia scale on the MMPI-2-rf, anxiety scale on the PAI). Scores on these scales were normed based on age; final scores on the subscales are presented as *T*-scores, which have a mean of 50 and a standard deviation of 10. Higher *T*-scores are indicative of more psychiatric symptomatology.

Given that some patients were administered the MMPI-2-rf while others were administered the PAI, several statistical tests were conducted to ensure that the subsamples which were given the MMPI versus the PAI did not differ in a systematic way. Chi-square tests or two-sample *t*-tests of the patient subsamples confirmed that demographic (i.e., age, gender, and level of education), treatment variables, or remission status did not differ significantly between the patients who received the MMPI-2-rf or the PAI. Further, the mean *T*-scores on each of the three emotional functioning constructs did not differ significantly in the group administered with PAI versus the group administered with MMPI-2-rf. Based on these analyses, the scores from both measures were combined into variables reflecting somatization, depression, and anxiety.

### 3.3. Statistical Methods

One-sample *t*-tests were conducted to determine whether *z*-scores were significantly different from 0. Significance was set at a *p* value < 0.05 (uncorrected). Cohen's *d* was also calculated to determine the magnitude of the effect size. We also tested whether pretreatment HPA hormone changes were related to neurocognitive functioning. Bivariate Pearson correlations were used to test the relationship between pretreatment ACTH levels and neurocognitive functioning, as well as the relationship between pretreatment urine free cortisol levels and neurocognitive functioning. Significance was set at a *p* value < 0.05 (uncorrected).

We also conducted two sample *t*-tests to determine whether there were any differences in neuropsychological or emotional functioning between individuals who had active Cushing's syndrome (*n* = 11) and patients who were in biochemical remission (*n* = 7). Given the small sample sizes, it was determined *a priori* that effect sizes (Hedges' *g*) would be used (rather than *p* values) and that only medium effect sizes (i.e., Hedges' *g* > 0.5) would be discussed.

Notably, this study could not examine the individual contribution of surgery or radiation on neuropsychological outcomes; the subsamples of individuals with surgery overwhelmingly also represented the individuals who also received radiation. Given the limited sample size, we could not statistically covary the effects of radiation on the analysis to isolate the deficits attributable by the presence of surgery.

## 4. Results

### 4.1. Subjective Cognitive and Emotional Complaints

The most common subjective cognitive complaint was in the domain of attention. With regard to emotional functioning, an increase in irritability/decreased frustration tolerance was most frequently endorsed. The frequency of subjective cognitive and emotional complaints is presented in Table 3.

### 4.2. Performance on Neuropsychological Measures

Mean group performance on each neuropsychological measure, and the results of the *t*-tests are presented in Table 4. The table also includes information on the number of individuals who met the criterion for impairment, which was defined as performance 1.5 standard deviations below the mean. This provides complementary information to group mean performance, as the mean statistic can be overly skewed by a minority of individuals with extremely low scores. Based on a normal distribution, we would expect that 7% of all healthy, normal adults would meet criterion for impairment. Knowing the frequency of the sample that meets the criterion for clinical impairment, particularly if the frequency greatly exceeds the expected 7%, provides additional support regarding domains of cognitive functioning that are frequently affected in patients with Cushing's syndrome.

Overall, simple attention and working memory were not significantly reduced compared to the normative sample. However, patients with CS were more inconsistent in their performance on sustained attention task. Processing speed was not significantly reduced. Performance on measures of visual-spatial skills was notable for significantly reduced spatial estimation of line angles; almost 30% of the sample was classified as clinically impaired on this measure. With respect to language, confrontation naming was not significantly reduced. Phonemic fluency was significantly above expectation compared to the normative sample. Performance was not significantly reduced on two tasks of cognitive flexibility.

Performance on visual-spatial learning and memory tasks was notable for significantly reduced recall following the first exposure of the stimuli. Total learning (when combining the performance across all three learning trials) was also significantly reduced. However, recall on trials 2 and 3, as well as the overall learning curve were not significantly different from zero, suggesting that reduced overall learning was largely due to poor learning on the first trial. Despite reduced learning, performance after a delay was not significantly lower than zero, suggesting that patients were successfully able to retrieve and retain the information that they learned on par with same-aged peers.

Similarly, performance on the verbal list learning task was notable for significantly reduced learning after the first exposure of the word list; 28% of the sample was classified as impaired on the first trial, suggesting that a significant proportion of the sample learn fewer words than expected after the first exposure of information. However, learning across the rest of the trials was not significantly reduced. Patients' ability to recall information following short and long delays was also within expectations. Overall, performance on both visual-spatial and verbal memory tasks was most notable for reduced learning upon first exposure of information. However, retention and retrieval of information learned was not significantly reduced.

### 4.3. Psychological Functioning

Mean *T*-scores on measures of emotional functioning are presented in Table 5. The measures are presented in *T*-scores, which have a mean of 50 and a standard deviation of 10; higher *T*-scores indicate higher levels of symptomatology. The table also includes information on the percentage of individuals in the sample who met criterion for impairment, which was defined as 2 standard deviations above the mean. Based on a normal distribution, we would expect that only 2% of all healthy, normal adults would endorse this level of symptomatology. Knowing the frequency of the sample that meets the criterion for clinical impairment, particularly if the frequency greatly exceeds the expected 2%, provides additional support regarding domains of emotional functioning that are frequently affected in patients with Cushing's syndrome.

Overall, patients with Cushing's syndrome endorsed significantly higher levels of somatization, higher levels of depression, and higher levels of anxiety than a normative sample. Over 50% of the sample endorsed clinically elevated levels of somatization and depressive symptomatology, whereas 22% of the sample endorsed clinically elevated levels of anxious symptomatology.

### 4.4. Correlations between Pretreatment HPA Hormone Levels and Neurocognitive Performance

Bivariate correlations were conducted between pretreatment ACTH levels and neurocognitive functioning. Results revealed significant relationships between pretreatment ACTH levels and poorer visual-spatial functioning (*r* = −0.55, *p* = 0.03), poorer confrontation naming (*r* = −0.69, *p* = 0.02), and poorer retention of verbal information after a short delay (*r* = −0.51, *p* = 0.04) and a longer delay (*r* = −0.62, *p* = 0.01). Bivariate correlations between pretreatment UFC levels and neurocognitive performance did not reveal any significant relationships.

### 4.5. Active Cushing's vs. Biochemical Remission

Performances on neuropsychological measures and emotional functioning measures were compared between patients with active Cushing's syndrome (*n* = 11) and patients in biochemical remission (*n* = 7). There were significant differences in the treatment between the two groups, as the patients in biochemical remission underwent surgical treatment in greater proportions than patients with active Cushing's syndrome (86% and 18%, respectively). The two groups did not differ significantly with respect to radiation treatment. As mentioned previously, it was determined *a priori* that effect sizes would be used rather than *p* values; only medium effect sizes or larger (Hedges' *g* > 0.5) will be discussed. Comparisons on all measures are available in the Supplemental Materials (S2).

Overall, patients with active Cushing's syndrome displayed lower processing speed; they performed significantly more poorly on Coding (*g* = 0.58) and Trails A (*g* = 0.81), and they were slower on the CPT (*g* = 0.86). Patients with active Cushing's syndrome also performed more poorly on an executive functioning task of cognitive flexibility (Trails B; *g* = 1.27), as well as a confrontation naming task (BNT; *g* = 0.52). Patients with active Cushing's disease made more omission errors (*g* = 0.78) and were more variable in their reaction times as the task progressed on a sustained attention task (*g* = 0.76). With regard to emotional functioning, patients with active Cushing's syndrome endorsed higher levels of somatization and anxiety compared to patients in biochemical remission (*g* = 0.58, 0.50, respectively).

## 5. Discussion

Results of this study indicated that patients with CS on average exhibited reduced sustained attention (particularly in the consistency of their attention) and reduced visuospatial functioning, particularly in the estimation of line angles. Learning and memory for both visual-spatial information and word lists were notable for reduced initial learning after the first exposure of stimuli, but intact retention of information learned. This pattern on learning and memory tasks is most suggestive of difficulties with encoding due to reduced or variable sustained attention, rather than memory deficits per se, as patients demonstrated good learning following multiple exposures to material, and intact retention of information learned. With regard to emotional functioning, patients with CS overwhelmingly endorsed high levels of somatization, increased levels of depression, and increased levels of anxiety. It is important to note that these findings are not exclusively due to a select minority of the sample who are skewing the group mean; between 20 and 30% of the sample meet the criterion for clinical impairment in the cognitive domains mentioned, and over 50% of the sample endorsed clinically elevated levels of somatization and depression.

Interestingly, inconsistencies in visual sustained attention (measured by a computerized continuous performance task) were found within the context of intact simple auditory attention on this study (as measured by a digit span task). Many other studies have exclusively used the digit span task as a measure of attention, and findings have been mixed as to whether attention deficits are present in patients with CS. Results of this study show that more sensitive measures of complex attention may need to be utilized to better understand the attention complaints with which patients present. Indeed, over 90% of our sample endorsed attention as an area that they have experienced subjective decline, but 0% of the sample met the criterion for impairments on the simple digit span attention task, suggesting that a simple digit span measure fails to fully capture the kinds of attention deficits that patients experience. It is important to note that the digit span task only requires that the patients attend to information for several seconds at a time; there are few tasks in daily life that require focused attention for such a limited span.

For both visuospatial and verbal information, patients exhibited the most difficulty with learning upon first exposure of the stimuli, and demonstrated improved performance when provided additional trials to learn the information, with intact retrieval and retention of the information that they learned. This type of memory profile is highly consistent with the idea that sustained attention difficulties interfere with initial encoding, but that patients are successfully able to consolidate the information that they learned and retrieve the information successfully after a long delay. This specific profile of memory functioning in patients may have been obscured in prior research studies investigating memory which used total learning scores that combine performance across multiple learning trials [4, 16]. Future studies should more specifically examine the role of complex attention in patients' ability to encode information on learning and memory tasks.

From a neurobiological perspective, the affected domains in this population localize to dysfunction of prefrontal regions and subcortical networks rather than dysfunction of the medial temporal lobe. This localization (based on neuropsychological data) supports current neuroimaging literature in this group (i.e., persistent structural and functional alterations in prefrontal regions). Future studies should examine the functional brain activity in patients with CS while they complete a sustained attention task in the scanner to elucidate the neurobiological processes underpinning complex attention deficits. Given that general performance metrics (e.g., accuracy and reaction time) on the task were not significantly reduced, it would be interesting to determine whether patients may be recruiting additional regions of the brain or activating the same regions at higher levels in order to compensate for their performance. This altered functional activity may even play a role in explaining the level of cognitive fatigue that patients regularly experience.

Findings of the study also suggest the need for targeted recommendations when patients complain of memory problems. It is important to educate patients about how memory relies on adequate encoding for information to be consolidated into long term storage and discuss how inconsistency in sustained attention may be interfering with encoding. Clinicians may provide more targeted recommendations for how to improve attention, including interventions to improve sustained attention (e.g., focused attention meditation training, computer-based cognitive training programs), as well as the use of self-talk strategies and the use of repetition [38]. It may also be important to educate patients about how attention is sensitive to a variety of lifestyle factors such as fatigue and lack of sleep; proactively managing these areas can help improve attention and memory. Pharmacological interventions to improve attention may also be an option and is an area for future research.

Another finding from this study is that patients struggled with some aspects of visual-spatial functioning, particularly in their spatial estimation of line angles. This finding has been replicated in several other studies [4]. It is our experience that patients with Cushing's often have functional complaints that may implicate compromised visual-spatial functioning (e.g., bumping into items in their environment, falling). Clearly this is an area that warrants further investigation. Indeed, findings from this study suggested that pretreatment ACTH levels were associated with poorer visual-spatial functioning, suggesting a link between glucocorticoid excess on this domain of functioning. In addition to visual-spatial functioning, pretreatment ACTH levels were also associated with poorer confrontation naming and poorer retention of verbal information. The association between higher ACTH levels and poorer memory is consistent with what is expected in cognition given hippocampal sensitivity to glucocorticoid excess.

The high rates of anxious and depressive distress are consistent with prior literature that has documented difficulties with emotional functioning in this group. This study also is one of the first to quantitatively document clinically elevated rates of somatization in this group. Elevated levels of somatization make sense within the context of patient's diagnostic history. Unfortunately, misdiagnosis is quite common in patients with Cushing's syndrome, and patients may be treated in a variety of other clinic settings before being accurately diagnosed [39]. Patients have also reported being dismissed by other medical providers prior to diagnosis. These issues, as well as fears of recurrence of the disease, may contribute to patients becoming preoccupied and hyperattentive to physical symptoms. Hyperattention to symptoms may extend to their perception of cognitive dysfunction. Patients' concerns about changes in their functioning, as well as fears of recurrence may contribute to heightened awareness of memory lapses that are entirely normative to the exclusion of other contradictory information which may indicate cognitive improvements or recovery. Given the high prevalence of emotional distress in this group, there is a clear need for continued follow-up of patients with CS; a multidisciplinary approach that involves mental health and psychiatry is warranted to optimize quality of life in these patients.

The findings from this study should be interpreted with caution in light of several limitations. First, this is a retrospective study and the sample is prone to selection bias. Patients were referred to the outpatient clinic largely when they brought up concerns about their cognitive or everyday functioning, suggesting that the sample may be skewed to overrepresent patients with cognitive or emotional problems and thus limits the generalizability of these findings. In addition, a control group was not used in this study, which increases concern regarding sources of systematic bias. A prospective research study with an appropriate control group is crucial to better understand the range and variability in cognitive and emotional outcomes in patients with CS. However, one strength of the study was in the use of standalone and embedded measures of performance validity, which provides additional support that scores on measures represent true areas of deficits rather than efforts to intentionally or unintentionally distort the cognitive profile.

Another limitation is the small sample size, which precluded a thorough examination of potential treatment (e.g., radiation) and comorbid medical factors (e.g., hypertension, diabetes mellitus) that may confer risk for poorer cognitive outcomes. For instance, patients in remission had either normal cortisol levels or treatment-related corticoadrenal insufficiency. Also, we did not control for other endocrinopathies that may occur in patients with Cushing's. For instance, prior research has found that secondary endocrinopathies such as hypogonadism and growth hormone deficiency has been associated with poorer cognition. There is a clear need for an increased sample size to fully investigate the individual impact of these treatment related factors and to control for potential confounds with more statistically sophisticated models. Given multiple possible pituitary abnormalities pre-and postoperatively (e.g., TSH, FSH, LH, and GH) and the fact that not all of our patients were on supplements at the time of testing, a larger sample size is necessary to explore these confounding effects on neuropsychological testing. Further, a larger sample size will allow for more detailed analyses to investigate how total hormonal changes are related to neurocognitive and psychological functioning patients with Cushing's syndrome. A research study is underway to recruit patients prospectively, and with an appropriate control group to isolate the cognitive deficits associated with sustained hypercortisolemia. Neuroimaging would be a valuable addition to this research to determine the extent of structural and functional changes (particularly related to changes in activation in the brain during sustained attention tasks) in the brain as a result of sustained hypercortisolemia.

Despite these limitations, we believe that our study also has several strengths. As mentioned previously, the use of measures that examine more complex areas of attention, as well as the examination of the entire memory profile, provided a more comprehensive picture of the cognitive deficits in this clinical group. This type of approach can lead to more precise recommendations to optimize day to day functioning. This study is also one of the first to investigate somatization in a quantitative fashion.

Given the high incidence of clinical elevations especially with somatization and depressive symptomatology, it will be crucial for future research to investigate the mechanism through which emotional difficulties persist, and to identify factors that could potentially moderate emotional functioning outcomes.

## 6. Conclusions

In this retrospective study, patients with CS on average exhibited reduced consistency of their sustained attention, reduced visuospatial functioning, and difficulties with initial learning after first exposure of stimuli (with intact retention of information learned). In clinical practice, more sensitive and ecologically valid measures of complex attention may need to be utilized to capture the types of attention difficulties that patients experience in their daily lives. Clinicians may also find it helpful to provide more targeted feedback for patients and discuss the possibility that difficulties with sustained attention may interfere with adequate encoding of information; recommendations for how to improve attention and interventions to improve sustained attention may prove to be useful.

This study also replicated prior findings of high rates of depressive and anxious symptomatology, indicating a clear need for continued follow-up of patients with CS, even for those who have achieved biochemical remission. A multidisciplinary approach that involves mental health and psychiatry is warranted to optimize quality of life in these patients. This study also found that patients with CS exhibited high levels of somatization, suggesting that emotional distress may play a role on cognitive functioning or the perception of cognitive dysfunction. Future research is needed to investigate the mechanism through which emotional difficulties persist, and to identify factors that could potentially moderate emotional functioning outcomes, such as coping styles and levels of social support.

## Figures and Tables

**Table 1:** Demographic, biochemical, and treatment details for Cushing's disease at the time of neurocognitive evaluation.

	Participants
	*n* = 18
Demographic information	
Females (*n*, %)	15 (83%)
Ethnicity (*n*, %)	
Caucasian	13 (72%)
African-American	5 (28%)
Mean age at examination (SD)	41.6 (10.6)
Range	25-70
Mean years of education (SD)	14.8 (2.7)
Range	10 - 18
IQ scaled score^∗^ (SD)	104 (11)
Biochemical information*^*	
Active Cushing's syndrome	11 (61%)
Biochemical remission	7 (39%)
Taking hydrocortisone replacement	3 (16.6%)
Treatment information	
No treatment	10 (55.5%)
Surgery alone	3 (16.6%)
Surgery followed by radiation (*n*, %)	4 (22%)
Pituitary radiation alone	1 (5.5%)
Median time from last treatment in months	38
Range	3-192

Note. ^∗^Intelligence was estimated using a validated measure of word reading and adjusted based on demographic information, including age, gender, ethnicity, and level of education [29]. ^Biochemical remission was defined as patients with either low or normal levels of cortisol, whereas patients with high levels of cortisol were categorized into the active Cushing's category. Patients with low cortisol as a result of pituitary or adrenal surgery were receiving hydrocortisone replacement. Of the 5 patients who had radiation, 3 had stereotactic radiosurgery and 2 fractionated stereotactic radiosurgery.

**Table 2:** Endocrinological and medical comorbidities.

Pretreatment endocrinological characteristics^∗^	
ACTH (pg/mL)	87.7 ± 41.8
Urine free cortisol (mcg/day)	383.4 ± 280.5
Pretreatment medical comorbidities (number of patients)^∗^	
Hypertension	12
Diabetes mellitus	9
Sleep apnea	1
Oligomenorrhea	3
Osteoporosis	5
Hypokalemia	2
Hirsutism	4
Heart failure	1
Male hypogonadism	3
Amenorrhea	4
Venous thromboembolic disease	1
Alopecia	1
Endocrine characteristics at the time of testing	
Potassium (mEq/L)	4.1 ± 0.4
Glucose (mg/dL)	109.1 ± 33.6
Hemoglobin A1c (%)^	6.3 ± 1.2
Posttreatment new secondary conditions	
Hypogonadism (*n*, %)	5 (28%)
Treatment at time of testing	20%
Hypothyroidism (*n*, %)	5 (28%)
Treatment at time of testing	100%
Growth hormone deficiency (*n*, %)	2 (11%)
Treatment at time of testing	50%

^∗^Mean ACTH levels are based on data from 16 patients, as one patient had missing information due to treatment at different clinic many years prior to the neurocognitive evaluation, while another patient had undetectable ACTH levels (as expected in adrenal Cushing's). Urine free cortisol levels and pretreatment medical comorbidities are based on data from 17 patients, as the same patient with missing ACTH level did not have the pretreatment information available. ^Mean hemoglobin A1c levels are based on data from 12 patients, while the remaining six patients did not have data available. However, blood glucose levels were reviewed for these patients to confirm that they did not have diabetes mellitus. Data is presented as mean ± standard deviation.

**Table 3:** Subjective cognitive and emotional complaints.

	Participants
Cognitive complaints	% (*n*)
Attention	94% (17)
Memory	78% (14)
Word finding	67% (12)
Processing speed	39% (7)
Emotional complaints	
Irritability	50% (9)
Anxiety	39% (7)
Sadness/low mood	39% (7)
Emotional liability	33% (6)

**Table 4:** Performance on neuropsychological measures.

Measure	*z*-scores		*n*	df	*t*	*p*	Cohen's *d*	% impaired^ (*n*)
	*M*	SD						
Simple attention								
Digit span forward	-0.31	0.81	18	17	0.12	0.12	-0.38	0% (0)
Digit span backward	-0.06	0.93	18	17	-0.29	0.77	-0.06	5.6% (1)
Sustained attention								
CPT omission errors	-0.40	1.68	15	14	-0.92	0.38	-0.24	20% (3)
CPT commission errors	-0.26	1.32	15	14	-0.75	0.47	-0.19	27% (4)
CPT hit reaction time	-0.55	1.27	15	14	-1.68	0.12	-0.43	20% (3)
CPT hit reaction time standard error	-0.76	1.39	15	14	-2.13	0.05^∗^	-0.55	20% (3)
CPT perseverations	-0.80	1.83	15	14	-1.69	0.11	-0.44	40% (6)
CPT hit reaction time block change	-0.54	0.90	15	14	-2.33	0.04^∗^	-0.60	13% (2)
CPT hit reaction time block change standard error	-0.84	0.81	15	14	-4.01	0.001	-1.03	20% (3)
Processing speed								
Trails A	0.35	0.78	12	11	1.55	0.15	0.45	0% (0)
Coding	-0.37	1.05	17	16	-1.45	0.17	-0.35	5.9% (1)
Visuospatial skills								
Facial recognition	-0.06	0.24	17	16	-1.0	0.33	-0.25	5.6% (1)
Judgment of line orientation	-0.71	1.05	17	16	-.28	0.01^∗∗^	-0.68	29% (5)
Language								
Boston naming test	-0.34	0.64	13	12	-1.9	0.08	-0.53	0% (0)
Verbal fluency phonemic	0.52	0.97	18	17	2.27	0.04^∗^	0.54	0% (0)
Verbal fluency category	0.17	1.11	18	17	0.63	0.54	0.15	5.6% (1)
Visuospatial memory								
BVMT trial 1	-0.95	1.09	15	14	-3.36	0.005^∗∗^	-0.87	26.7% (4)
BVMT trial 2	-0.66	1.53	15	14	-1.67	0.12	-0.43	33.3% (5)
BVMT trial 3	-0.56	1.27	15	14	-1.7	0.11	-0.44	26.7% (4)
BVMT total recall (trials 1-3)	-0.83	1.33	15	14	-2.43	0.03^∗^	-0.62	33.3% (5)
BVMT learning	0.51	1.06	15	14	1.85	0.09	0.48	0% (0)
BVMT delayed recall	-0.54	1.43	15	14	-1.47	0.16	-0.38	26.7% (4)
Verbal memory								
CVLT trial 1	-0.81	1.05	18	17	-3.33	0.005^∗∗^	-0.77	27.8% (5)
CVLT trial 2	-0.34	0.99	18	17	-1.47	0.16	-0.34	16.7% (3)
CVLT trial 3	-0.36	1.29	18	17	-1.19	0.25	-0.28	16.7% (3)
CVLT trial 4	-0.25	1.23	18	17	-.86	0.4	-0.20	16.7% (3)
CVLT trial 5	-0.23	.93	18	17	-1.1	0.31	-0.25	16.7% (3)
CVLT total learning (trials 1-5)	-0.17	1.00	18	17	-0.72	0.48	-0.17	11% (2)
CVLT list B	-0.59	0.72	18	17	-4.03	0.001^∗∗^	-0.82	22.2% (4)
CVLT short delay free recall	-0.21	1.21	18	17	-0.74	0.47	-0.17	11.1% (2)
CVLT short delay cued recall	-0.12	1.07	18	17	-0.48	0.64	-0.11	16.7% (3)
CVLT long delay free recall	-0.15	1.18	18	17	-0.54	0.60	-0.13	11.1% (2)
CVLT long delay cued recall	-0.13	1.22	18	17	-0.45	0.66	-0.11	22.2% (4)
CVLT recognition hits	-0.36	1.22	18	17	-1.25	0.23	-0.3	16.7% (3)
CVLT false positives	-0.04	1.23	18	17	-0.13	0.90	-0.03	0% (0)
Executive functioning								
Trails B	-.01	1.05	12	11	-.03	.97	-0.01	16.7% (2)
Verbal fluency switching	.63	1.17	18	17	2.3	.03∗	0.54	0% (0)

*Note*. ^∗^*p* < 0.05 (uncorrected), ^∗∗^*p* < 0.01. ^Impairment was defined as 1.5 standard deviations below the normative mean.

**Table 5:** Psychological functioning.

Measure	*T*-scores		*n*	df	*t*	*p*	Effect size	% clinically elevated^ (*N*)
	*M*	SD						
Somatization	73.88	13.41	17	16	7.35	0.00^∗∗^	1.78	59% (10)
Depression	69.00	14.16	17	16	5.53	0.00^∗∗^	1.34	59% (10)
Anxiety	62.12	11.95	17	16	4.18	0.001^∗∗^	1.01	22% (4)

*Note*. ^∗^*p* < 0.05 (uncorrected), ^∗∗^*p* < 0.01. ^Clinical elevation was defined as 2 standard deviations above the mean (i.e., *T* > 70).

## Data Availability

Data are available from the corresponding author upon reasonable request.

## Supplementary Materials

Supplementary Materials S1: detailed descriptions of the neuropsychological measures included in the battery. S2: a table consisting of comparisons of mean neuropsychological and emotional functioning in patients with active Cushing's vs. patients in biochemical remission

## References

[1] Lambert J. K., Goldberg L., Fayngold S., Kostadinov J., Post K. D., Geer E. B. (2013). Predictors of mortality and long-term outcomes in treated Cushing's disease: a study of 346 patients. *The Journal of Clinical Endocrinology and Metabolism*.

[2] Newell-Price J., Bertagna X., Grossman A. B., Nieman L. K. (2006). Cushing's syndrome. *Lancet*.

[3] Ioachimescu A. G. (2018). Prognostic factors of long-term remission after surgical treatment of Cushing's disease. *Endocrinology and Metabolism Clinics of North America*.

[4] Frimodt-Moller K. E., Mollegaard Jepsen J. R., Feldt-Rasmussen U., Krogh J. (2019). Hippocampal volume, cognitive functions, depression, anxiety, and quality of life in patients with Cushing syndrome. *The Journal of Clinical Endocrinology and Metabolism*.

[5] Dorn L. D., Burgess E. S., Friedman T. C., Dubbert B., Gold P. W., Chrousos G. P. (1997). The longitudinal course of psychopathology in Cushing's syndrome after correction of hypercortisolism. *The Journal of Clinical Endocrinology & Metabolism*.

[6] Forget H., Lacroix A., Cohen H. (2002). Persistent cognitive impairment following surgical treatment of Cushing's syndrome. *Psychoneuroendocrinology*.

[7] Whelan T. B., Schteingart D. E., Starkman M. N., Smith A. (1980). Neuropsychological deficits in Cushing's syndrome. *The Journal of Nervous and Mental Disease*.

[8] Dorn L. D., Burgess E. S., Dubbert B. (1995). Psychopathology in patients with endogenous Cushing's syndrome: 'atypical' or melancholic features. *Clinical Endocrinology*.

[9] Sonino N., Fava G. A. (1998). Psychosomatic aspects of Cushing's disease. *Psychotherapy and Psychosomatics*.

[10] Starkman M. N., Schteingart D. E., Schork A. M. (1981). Depressed mood and other psychiatric manifestations of Cushing's syndrome: relationship to hormone levels. *Psychosomatic Medicine*.

[11] Haskett R. F. (1985). Diagnostic categorization of psychiatric disturbance in Cushing's syndrome. *The American Journal of Psychiatry*.

[12] Hudson J., Hudson M. S., Griffing G. T., Melby J. C., Pope H. G. (1987). Phenomenology and family history of affective disorder in Cushing's disease. *The American Journal of Psychiatry*.

[13] Broersen L. H. A., Andela C. D., Dekkers O. M., Pereira A. M., Biermasz N. R. (2019). Improvement but no normalization of quality of life and cognitive functioning after treatment for Cushing’s syndrome. *The Journal of Clinical Endocrinology and Metabolism*.

[14] Tiemensma J., Biermasz N. R., Middelkoop H. A. M., van der Mast R. C., Romijn J. A., Pereira A. M. (2010). Increased prevalence of psychopathology and maladaptive personality traits after long-term cure of Cushing's disease. *The Journal of Clinical Endocrinology and Metabolism*.

[15] Conrad C. D. (2008). Chronic stress-induced hippocampal vulnerability: the glucocorticoid vulnerability hypothesis. *Reviews in the Neurosciences*.

[16] Tiemensma J., Kokshoorn N. E., Biermasz N. R. (2010). Subtle cognitive impairments in patients with long-term cure of Cushing's disease. *The Journal of Clinical Endocrinology and Metabolism*.

[17] Bourdeau I., Bard C., Forget H., Boulanger Y., Cohen H., Lacroix A. (2005). Cognitive function and cerebral assessment in patients who have Cushing's syndrome. *Endocrinology and Metabolism Clinics of North America*.

[18] Pires P., Santos A., Vives-Gilabert Y. (2017). White matter involvement on DTI-MRI in Cushing's syndrome relates to mood disturbances and processing speed: a case-control study. *Pituitary*.

[19] Andela C. D., van der Werff S. J. A., Pannekoek J. N. (2013). Smaller grey matter volumes in the anterior cingulate cortex and greater cerebellar volumes in patients with long-term remission of Cushing's disease: a case-control study. *European Journal of Endocrinology*.

[20] Hou B., Gao L., Shi L. (2020). Reversibility of impaired brain structures after transsphenoidal surgery in Cushing's disease: a longitudinal study based on an artificial intelligence-assisted tool. *Journal of Neurosurgery*.

[21] Jiang H., He N. Y., Sun Y. H. (2017). Altered gray and white matter microstructure in Cushing's disease: a diffusional kurtosis imaging study. *Brain Research*.

[22] van der Werff S. J. A., Andela C. D., Nienke Pannekoek J. (2014). Widespread reductions of white matter integrity in patients with long-term remission of Cushing's disease. *NeuroImage: Clinical*.

[23] Bas-Hoogendam J. M., Andela C. D., van der Werff S. J. A. (2015). Altered neural processing of emotional faces in remitted Cushing's disease. *Psychoneuroendocrinology*.

[24] Langenecker S. A., Weisenbach S. L., Giordani B. (2012). Impact of chronic hypercortisolemia on affective processing. *Neuropharmacology*.

[25] Ragnarsson O., Stomby A., Dahlqvist P. (2017). Decreased prefrontal functional brain response during memory testing in women with Cushing's syndrome in remission. *Psychoneuroendocrinology*.

[26] Stomby A., Salami A., Dahlqvist P. (2019). Elevated resting-state connectivity in the medial temporal lobe and the prefrontal cortex among patients with Cushing's syndrome in remission. *European Journal of Endocrinology*.

[27] Petersen S. E., Posner M. I. (2012). The attention system of the human brain: 20 years after. *Annual Review of Neuroscience*.

[28] Sonino N., Fallo F., Fava G. A. (2010). Psychosomatic aspects of Cushing's syndrome. *Reviews in Endocrine and Metabolic Disorders*.

[29] Holdnack H. A. (2001). *Wechsler Test of Adult Reading: WTAR*.

[30] Wechsler D. (2008). *Wais-IV Administration and Scoring Manual*.

[31] Conners C. K. (2004). *Conners' Continuous Performance Test*.

[32] Benton A. L., Hamsher K., Varney N. R., Spreen O. (1983). *Contributions to Neuropsychological Assessment*.

[33] Kaplan E., Goodglass H., Weintraub S. (1983). *Boston Naming Test*.

[34] Delis D. C., Kaplan E., Kramer J. H. (2001). *Delis-Kaplan Executive Function System*.

[35] Delis D. C., Kramer J. H., Kaplan E., Ober B. A. (2000). *California Verbal Learning Test - Second Edition. Adult Version. Manual*.

[36] Ben-Porath Y. S., Tellegen A. (2011). *MMPI-2-RF (Minnesota Multiphasic Personality Inventory-2-Restructured Form): Manual for Administration, Scoring, and Interpretation*.

[37] Morey L. C. (2007). *Personality Assessment Inventory Professional Manual*.

[38] Fortenbaugh F. C., Rothlein D., McGlinchey R., DeGutis J., Esterman M. (2018). Tracking behavioral and neural fluctuations during sustained attention: a robust replication and extension. *NeuroImage*.

[39] Boscaro M., Barzon L., Sonino N. (2000). The diagnosis of Cushing's syndrome. *Archives of Internal Medicine*.

