# Supplementary material for: Neuropsychological and Emotional Functioning in Patients with Cushing's Syndrome
Source: Behav Neurol. 2020 Aug 7;2020:4064370. doi: 10.1155/2020/4064370 (PMC7428886; doi:10.1155/2020/4064370)
Supplement: Supplementary Materials — S1: detailed descriptions of the neuropsychological measures included in the battery. S2: a table consisting of comparisons of mean neuropsychological and emotional functioning in patients with active Cushing's vs. patients in biochemical remission [file 4064370.f1.docx]

**Supplemental Information**

S1. Description of neuropsychological measures

Cognitive Functioning

*Simple auditory attention and working memory*. Simple auditory attention and working memory were assessed using the Digit Span subtest of the Wechsler Adult Intelligence Scale—Fourth Edition. On this measure, patients were presented with a string of digits and asked to repeat them back exactly as they heard them to the examiner. The Digit Span Forward metric is the number of digits patients correctly repeat back to the examiner. Patients were then given a string of numbers and asked to repeat them back to the examiner in backwards order. The digits backward metric is the number of digits they were able to correctly repeat in the backwards order, and is a measure of auditory working memory.

*Sustained attention*. Sustained attention was assessed using the Conners’ Continuous Performance Test, 2^nd^ edition. This measure is a fifteen-minute computerized task where patients are shown a series of letters and instructed to press the space bar whenever they see a letter with the exception of one target letter. This measure yields metrics that reflect the patient’s accuracy (omission and commission errors), reaction time, and consistency (standard error of reaction times, changes in reaction time, as well as changes in standard errors as the task progresses).

*Processing Speed*. This domain was assessed with two tasks: Trails A and the Coding subset from the WAIS-IV. On Trails A, patients are presented with a sheet of paper with numbers in various locations on the page, and asked to sequence the numbers in order as quickly as possible. Performance on this measure is based on the amount of time the patient takes to finish the entire task accurately. The Coding subtest is a symbol transcription task, and performance on this measure is based on the number of items that the patient completes within a two-minute span.

*Visuospatial Skills*. This domain was assessed with two tasks: Judgment of Line Orientation and the Benton Facial Recognition task. On the judgment of line orientation measure, patients were asked to match two angled lines to a set of 11 lines arranged in a semicircle. The correct number of items are tallied and undergo correction for age and gender. On the Benton Facial Recognition task, patients are presented with unfamiliar faces and asked to match the face among a set of increasingly challenging foils. Performance on this measure is based on the number of correct items.

*Language*. Confrontation naming, the ability to retrieve the correct name of familiar and nonfamiliar objects, was assessed using the Boston Naming Test-II. On this measure, patients are shown a series of black and white line drawings. Performance on this measure is based on the number of items that patients are able to spontaneously name. Patients are also given credit for items correctly named with a semantic cue, which is only given if they clearly misperceive the image. Verbal fluency, the ability to quickly come up with words based on cues, was assessed using the Verbal Fluency subtest of the Delis-Kaplan Executive Function System. This measure yields several metrics: phonemic fluency is based on the number of words that patients can name that start with a certain letter of the alphabet in 60 seconds, and semantic fluency is based on the number of words that patients can name that belong to a certain semantic category in 60 seconds.

*Visuospatial Learning and Memory*. This domain was assessed using the Brief Visuospatial Memory Test-Revised. On this measure, patients are presented with an array of six geometric figures for ten seconds at a time. The stimuli is removed, and the patient is then asked to draw as much of the figures on a blank piece of paper. Patients are presented the images and asked to draw the images three separate times (Trials 1, 2, 3). Performance on these trials reflects the information that patients are able to successfully encode during the learning trials. After a twenty-five minute delay, the patients are asked to recall as much of the figures on their own; performance on this trial reflects their free retrieval/retention of information learned. Patients are given credit for the accuracy of their drawings as well as the accuracy of the locations of the drawings on the page. Finally, the patients are given a recognition trial, where they are presented the six original images among six foils and asked to correctly identify which of the figures were on the original display.

*Verbal Learning and Memory*. This domain was assessed using the California Verbal Learning Test-2^nd^ edition. In this measure, a list of sixteen words is presented to the patient, and they are asked to immediately recall as many words as they can remember. The list is presented a total of five times, and their recall after presentations of the word list reflects verbal learning. Patients are then immediately presented with a distractor word list (List B), then asked to recall as many words as they can freely (short delay free recall), and with semantic cues (short delay cued recall). After a twenty-minute delay, patients are again asked to recall as many words as they can freely (long delay free recall), and then with semantic cues (long delay cued recall). Finally, patients are given a recognition trial, where they are presented with the 16 words from the original list alongside 16 foils and asked to correctly identify the words that were presented on the initial word list.

*Executive Functioning.* Executive functioning was assessed using Trails B and the Switching trial of the Verbal Fluency subtest of the DKEFS. On Trails B, patients are presented with a piece of paper with numbers and letters, and asked to alternate in order between numbers and letters. Performance on this measure is based on the amount of time the patient takes to finish the entire task accurately. On the Verbal Fluency Switching trial, patients are given two semantic categories and told to alternate between the two categories when giving responses. Performance is assessed based on the number of accurate switches between categories within sixty seconds.

S2. Comparisons of neuropsychological and emotional functioning in patients with Active Cushing’s vs. patients in biochemical remission

| Measure | Biochemical Remission | | Active Cushing’s | | t | p | Hedges’ g |
| --- | --- | --- | --- | --- | --- | --- | --- |
|  | Mean Z-Scores | *SD* | Mean Z-Scores | SD |  |  |  |
| Digit Span Forward | -0.18 | 1.11 | -0.39 | 0.59 | 0.52 | 0.61 | 0.24 |
| Digit Span Backward | 0.06 | 1.23 | -0.15 | 0.73 | 0.46 | 0.65 | 0.21 |
| CPT Omission Errors | 0.49 | 0.51 | -0.84 | 1.91 | 1.51 | 0.16 | 0.78 |
| CPT Commission Errors | -0.22 | 1.12 | -0.27 | 1.46 | 0.07 | 0.95 | 0.03 |
| CPT Hit Reaction Time | 0.18 | 0.56 | -0.92 | 1.39 | 1.67 | 0.12 | 0.86 |
| CPT Hit Reaction Time Standard Error | -0.05 | 1.10 | -1.12 | 1.42 | 1.48 | 0.16 | 0.76 |
| CPT Perseverations | -0.38 | 1.08 | -1.00 | 2.13 | 0.61 | 0.55 | 0.31 |
| CPT Hit Reaction Time Block Change | -0.76 | 1.20 | -0.43 | 0.76 | -0.65 | 0.53 | -0.33 |
| CPT Hit Reaction Time Block Change Standard Error | -0.67 | 0.84 | -0.92 | 0.82 | 0.54 | 0.60 | 0.28 |
| Trails A | 0.73 | 1.02 | 0.08 | 0.48 | 1.51 | 0.16 | 0.81 |
| Trails B | 0.70 | 0.85 | -0.51 | 0.90 | 2.35 | 0.04 | 1.27 |
| Coding | 0.00 | 1.23 | -0.63 | 0.88 | 1.23 | 0.24 | 0.58 |
| Facial Recognition | 0.00 | 0.00 | -0.09 | 0.30 | 0.73 | 0.48 | 0.35 |
| Judgment of Line Orientation | -0.50 | 0.84 | -0.82 | 1.17 | 0.59 | 0.57 | 0.28 |
| Boston Naming Test | -0.12 | 0.64 | -0.48 | 0.65 | 0.99 | 0.34 | 0.52 |
| Verbal Fluency Phonemic | 0.43 | 0.71 | 0.58 | 1.14 | -0.31 | 0.76 | -0.14 |
| Verbal Fluency Category | 0.43 | 1.15 | 0.00 | 1.12 | 0.78 | 0.44 | 0.36 |
| Verbal Fluency Switching | 0.62 | 1.35 | 0.64 | 1.10 | -0.03 | 0.97 | -0.02 |
| BVMT Trial 1 | -1.14 | 0.78 | -0.85 | 1.25 | -0.47 | 0.65 | -0.24 |
| BVMT Trial 2 | -0.16 | 2.03 | -0.91 | 1.27 | 0.89 | 0.39 | 0.46 |
| BVMT Trial 3 | -0.34 | 1.74 | -0.67 | 1.06 | 0.46 | 0.65 | 0.24 |
| BVMT Total Recall (Trials 1-3) | -0.54 | 1.70 | -0.98 | 1.18 | 0.59 | 0.56 | 0.30 |
| BVMT Learning | 0.66 | 1.07 | 0.43 | 1.11 | 0.38 | 0.71 | 0.20 |
| BVMT Delayed Recall | -0.48 | 1.84 | -0.57 | 1.28 | 0.11 | 0.91 | 0.06 |
| CVLT Trial 1 | -1.00 | 0.50 | -0.68 | 1.29 | -0.62 | 0.55 | -0.28 |
| CVLT Trial 2 | -0.57 | 0.61 | -0.20 | 1.17 | -0.77 | 0.45 | -0.36 |
| CVLT Trial 3 | -0.36 | 0.63 | -0.36 | 1.61 | 0.01 | 0.99 | 0.00 |
| CVLT Trial 4 | -0.36 | 0.80 | -0.18 | 1.47 | -0.29 | 0.78 | -0.13 |
| CVLT Trial 5 | -0.50 | 1.00 | -0.06 | 0.89 | -0.97 | 0.34 | -0.45 |
| CVLT Total Learning (Trials 1-5) | -0.36 | 0.44 | -0.05 | 1.24 | -0.63 | 0.54 | -0.29 |
| CVLT List B | -0.57 | 0.53 | -0.76 | 0.83 | 0.52 | 0.61 | 0.24 |
| CVLT Short Delay Free Recall | -0.36 | 1.14 | -0.12 | 1.30 | -0.39 | 0.70 | -0.18 |
| CVLT Short Delay Cued Recall | -0.29 | 1.04 | -0.02 | 1.13 | -0.51 | 0.62 | -0.24 |
| CVLT Long Delay Free Recall | -0.21 | 0.86 | -0.11 | 1.38 | -0.18 | 0.86 | -0.08 |
| CVLT Long Delay Cued Recall | -0.43 | 1.06 | 0.06 | 1.33 | -0.82 | 0.42 | -0.38 |
| CVLT Recognition Hits | -0.50 | 1.12 | -0.27 | 1.33 | -0.37 | 0.71 | -0.17 |
| CVLT False Positives | -0.14 | 0.63 | 0.03 | 1.52 | -0.28 | 0.78 | -0.13 |

|  | Biochemical Remission | | Active Cushing’s | | t | p | Hedge’s g |
| --- | --- | --- | --- | --- | --- | --- | --- |
|  | Mean T-Scores | *SD* | Mean T-Scores | SD |  |  |  |
| Somatization | 68.67 | 14.19 | 76.73 | 12.71 | -1.20 | 0.25 | -0.58 |
| Depression | 73.17 | 16.62 | 66.73 | 12.92 | 0.89 | 0.39 | 0.43 |
| Anxiety | 58.17 | 9.97 | 64.27 | 12.81 | -1.01 | 0.33 | -0.50 |
